# Semantic Distances in Depression: Relations Between ME and PAST, FUTURE, JOY, SADNESS, HAPPINESS

**DOI:** 10.1007/s10936-016-9442-2

**Published:** 2016-06-23

**Authors:** Marlena Bartczak, Barbara Bokus

**Affiliations:** Faculty of Psychology, University of Warsaw, Stawki 5/7, 00-183 Warsaw, Poland

**Keywords:** Depression, Remission, Concepts, Notions, Semantic distance

## Abstract

Using the Semantic Distance Task, we investigated the semantic distances between ME and five metaphorically conceptualized notions: PAST, FUTURE, JOY, SADNESS, and HAPPINESS. Three Polish-speaking groups participated in the study: depressive subjects ($n = 30$), patients in remission ($n = 12$), and non-depressed individuals ($n = 30$). *T*-test and the Kruskal–Wallis nonparametric equivalent of ANOVA analyses showed that subjects in remission placed ME significantly farther away from PAST than non-depressed individuals and depressed patients. Data mining algorithms indicated the distances ME–SADNESS, ME–PAST, and ME–FUTURE as the three strongest predictors of group membership. We interpret the findings in the light of a contrast effect and defense mechanisms. We propose that intergroup differences are especially prominent in tasks requiring creation of semantic associative relations, that is, in the first stage of conceptual processing. We suggest treating the results as confirmation that Beck’s theory of depression applies at the level of notion comprehension, proving that processing of key concepts in depression symptoms (particularly PAST) runs differently in all three groups under consideration.

## How Depression Influences Production of Metaphorical Conceptualizations of Notions

The problem of how patients with depression, and those in remission from depression, understand *notions* (interpreted in this paper as mental representations of elements of the external or inner reality) has seldom been the subject of empirical research. Beck’s theory of depression (Beck 1963, 1967), a cognitive theory of depression that is among the best-known, and most often corroborated empirically (cf. Solomon and Haaga 2005), places the main emphasis on cognitive disorders in thinking. In particular, it describes dysfunctional thinking patterns and negative automatic thoughts that focus mainly on three areas of experience: the self (me), the personal future, and the world (the *cognitive triad*; cf. Beck 1995). The processing of notions has only a minor presence in Beck’s theory. His *content-specificity hypothesis *(Beck 1976) states that negative stimuli—including verbal ones—attract the attention of depressed subjects particularly strongly (for empirical studies, see e.g., Blaut 2003; Fales et al. 2008; Gollan et al. 2008; Lamberton and Oei 2008; for a review, see e.g., Gotlib and Neubauer 2000).

Meanwhile, there is much evidence to suggest that depression disturbs the mechanism of notion comprehension, in particular affecting metaphorical processing. Studies on depressive patients provide evidence that several cognitive functions are disrupted as a result of depression, including those that play a key role in metaphorical processing. They are mainly executive and attentional functions and working memory.

Poorer executive functions, and especially executive control deficits, are one of the most often replicated results on the cognitive functioning of depressed subjects (Holmes and Pizzagalli 2007; Joorman et al. 2006; see also Dichter et al. 2009: fMRI evidence for problems with cognitive control). Problems with executive functions in depression manifest themselves mainly in tasks requiring monitoring of execution and flexible changes of behavior (e.g., Siegle et al. 2004). One of the key causes of disruption in processes requiring control and concentration in depression are changes in patients’ attentional functioning (e.g., Kemp et al. 2009; Mahurin et al. 2006; Smith et al. 2006; for a review, see Georgieff et al. 1998). Some explanations refer to the classical theory that accounts for the contribution of affective states to the distribution of attentional resources (Ellis and Ashbrook 1988; for a review, see e.g., Olofsson et al. 2008). Others invoke the fact that people with depression dedicate a lot of their cognitive resources to processing information related to mistakes (or information related to depressive mood, see e.g., Stordal et al. 2004; or—more generally—information irrelevant to the task in hand, see e.g., Hecker and Meiser 2005), which reduces their capacity for effective cognitive functioning. Another key area of cognitive functioning that is disrupted by depression is memory (for a review, see e.g., Ellwart et al. 2003). In particular, many studies (e.g. Fossati et al. 1999; Hecker and Sędek 1999; but see also Harvey et al. 2005) suggest that depressed subjects have deficits of working memory (in the sense of Engle’s model; Engle et al. 1999; which is an interesting attempt to merge attention and memory theory, and emphasizing individual differences in working memory). One possible explanation is that a person’s negative mood in itself engages the cognitive resources too much, inclining the individual to remember and process information that is compatible with that mood (Dalgleish et al. 2007; Stordal et al. 2004; for a review, see Piotrowski and Wierzchoń 2009) though not necessarily relevant to the cognitive task at hand (cf. also studies on depressive ruminations; e.g., Joormann and Gotlib 2008; Levens et al. 2009). Recently it has often been emphasized that cognitive deficits in depressive subjects are revealed especially during effortful, elaborative processing, but not when automatic and pre-attentional processes are involved (cf. the “integrated theory,” Williams et al. 2007; reviewed in Ellwart et al. 2003; cf. also the cognitive exhaustion model, Sędek et al. 2010; for empirical support, see e.g., Ellwart et al. 2003; Sędek and Hecker 2004).

Results of research on how depression-induced cognitive changes subside during remission are inconsistent. Earlier publications suggest that they recede with the remission of depression symptoms (e.g., Barnett and Gotlib, 1988, as cited in Ilardi and Craighead 1999). But more recent research indicates that even people cured of depression present a special pattern of information processing (Atchley et al. 2007; Biringer et al. 2005; Holmes and Pizzagalli 2007; Neu et al. 2005). Recently, it has even been suggested that the root of cognitive vulnerability to depression could be genetic predisposition, and that this negative bias may already manifest itself in early childhood (Hayden et al. 2008). Ruminations (i.e., automatic, uncontrolled negative thoughts about oneself, the world, and the future) are suggested to be one possible mechanism of this influence (Joormann and Gotlib 2008). Ruminations are often associated with depressive individuals’ problems with attention, and with controlling working memory content (Joormann and Gotlib 2008; cf. also Levens et al. 2009), and are considered a factor in susceptibility to depression and in recurrence of depressive episodes (Nolen-Hoeksema, 2000; Nolen-Hoeksema and Larson, 1999, as cited in Joormann and Gotlib 2008).

Cognitive functions that are disturbed as a result of depression also play a major role in the production and comprehension of metaphors (in particular, working memory; e.g., Chiappe and Chiappe 2007; Monetta and Pell 2007; and supression processes, e.g., Gernsbacher et al. 2001). Furthermore, there is considerable research (e.g., reviewed in Talarowska et al. 2011) pointing to the existence of close neurobiological links between mood, attention, and figurative language processing (in particular, the role of the right hemisphere). An especially important role in metaphorical processing appears to be played by working memory. This is confirmed by theoretical models, e.g., Kintsch’s (2000, 2001) predication model (reviewed, e.g., in Chiappe and Chiappe 2007) or Glucksberg’s class-inclusion model (Glucksberg 2001, 2003; Glucksberg and Keysar 1990). The Kintsch model implies that people with working memory deficits (a) can have insufficient resources to activate an adequately developed semantic network, and (b) are less able to suppress the distinctive, but irrelevant, features of the predicate, due to which they usually take longer to provide an interpretation of a metaphorical statement, and their interpretations are of poorer quality (cf. also Blasko 1999; Gernsbacher et al. 2001). Glucksberg’s model assumes a substantial role of working memory mechanisms and executive functions (especially control and suppression) in the correct interpretation of metaphorical statements. According to Glucksberg, interpreting a metaphor (e.g., *Cigarettes are a time bomb*) requires one to create an *ad hoc* context-appropriate higher category. Those features of the metaphor carrier that are key to the metaphor’s meaning are highlighted (e.g., *health * and *life hazard*), while those that apply to its basic category, but are unimportant for the metaphor’s meaning (e.g., *a tool of terrorist activity*), are suppressed. The role of these mechanisms (also known as *priming *and *suppression effects*), especially suppression, have been confirmed by research, including that of Gernsbacher and associates from 2001. The importance of working memory in metaphorical processing has been confirmed by many empirical studies (e.g., Chiappe and Chiappe 2007; Monetta and Pell 2007).

## Results of Earlier Research on Metaphor Processing by Depressive Subjects

The similarity between cognitive functions that are disturbed as a result of depression, and those that are significant from the point of view of metaphorical processing, prompted us to conclude (see Bartczak and Bokus 2015) that depression could be correlated with changes in cognitive representations of notions; in particular that (a) depressive subjects would produce fewer metaphors of a given notion than healthy subjects, and that (b) compared to the cognitive representations of notions produced by healthy subjects, the cognitive representations of neutral and positive notions produced by depressive subjects would have more negative valence, while the cognitive representations of negative notions - more positive valence. (We understood *notions* as mental representations of elements of the external or inner reality. In our study, *notions* are not beliefs but mental representations of the signified elements of linguistic signs. Thus, notions are “concepts in use.”) We also assumed that a depressive pattern of cognitive representation would be observable during remission of the disorder. We adopted concepts that are of key importance from the point of view of depression itself and its symptoms (PAST, FUTURE, JOY, SADNESS, and HAPPINESS^1^). Three adult Polish-speaking groups participated in the study: patients suffering from depression, those currently in remission from depression, and non-depressed individuals. The task (the Questionnaire of the Metaphorical Conceptualization of a Notion, QMCN; for a detailed description, see Bartczak and Bokus 2015) was to read sentences about PAST, FUTURE, JOY, SADNESS, and HAPPINESS and to assess how accurately they described the notions. Each sentence had previously been judged by competent raters relative to valence, metaphoricity, and conventionalization. (For specimen sentences and detailed information about participants, data analysis, and results, see Bartczak and Bokus 2015).

Contrary to our predictions, the results did not confirm that depressive subjects have problems with processing metaphorical content. We interpret this as supporting:

1. Theories that posit the automatic, unconscious, and effortless character of metaphorical content processing;
2. Votes claiming that the process of producing and understanding metaphors occurs as automatically and rapidly as with literal statements (e.g., Gernsbacher et al. 2001; Glucksberg 2003);
3. Neuropsychological theories of metaphor (e.g., Schnitzer and Pedreira 2005) assuming that metaphorical thinking is natural for humans, and results from the way the brain is organized and operates;
4. Models predicting that depressive cognitive deficits emerge only in processing that requires effort, and not in automatic and unconscious processes (cf. Williams’ integrated theory, Williams, 1988, 1997, cited in Ellwart et al. 2003; cf. also the theory of Sȩdek invoking the cognitive exhaustion model, for a review, see Sędek et al. 2010).

On the other hand, the valence prediction has found strong confirmation in the results. We see this as evidence that the negative cognitive patterns posited by Beck (1963, (1967) also manifest themselves at the level of notions comprehension. One possible explanation for a negative interpretation bias in depressive subjects is the influence that ruminations have on cognitive processes. Ruminations are activated, among other things, by a negative mood, and last for a long time due to factors such as depressive subjects’ deficits in refreshing their working memory (Joormann and Gotlib 2008). It is possible that the high activity of negative representations, and problems with replacing them with positive ones leading to mood improvement, is conducive to negative processing of new stimuli, including verbal ones (for results concerning nonverbal stimuli, see e.g., Gollan et al. 2008).

Contrary to our expectations (and the results of other studies, e.g., Atchley et al. 2007; Holmes and Pizzagalli 2007; Watkins et al. 2000), the prediction as to the “depressive” model of replies given by subjects in remission from depression was not confirmed by the results. An analysis of the results for three groups showed that all the statistically significant correlations observed between the results of the depressed group and the group in remission were also found between the depressed group and the healthy group. This is all the more interesting is that subjects in remission from depression gave almost the same replies as subjects from the control group, and significantly different ones from those of patients suffering from depression. The first interpretation that springs to mind is that cognitive changes during remission simply subside (for a similar claim, see e.g., Barnett and Gotlib, 1988, cited in Ilardi and Craighead 1999). Interestingly, however, when we took valence into account, the replies of subjects in remission in several cases were even more “non-depressive” (had more positive valence) than the replies of healthy subjects. The question arises: Does a depressive episode in the past act like a vaccine, “retuning” conceptual mechanisms in a non-depressive direction (e.g., by activating positively marked semantic networks)? Or is this result, perhaps, the effect of defense mechanisms that replace negative representations with positive ones (cf. the case of PAST) and thus enable individuals to function effectively? The proposed interpretation does not dispel all doubts, e.g., how do we explain the tendency, observed in some patients with a depressive episode in their case history, for recurrences of depression in the future? Of course we cannot preclude another interpretation, i.e., that cognitive changes caused by depression continue (though less intensively) in remission from depression, but are not apparent in tasks requiring metaphorical sentence processing.

## Studying the Relational Network Between Notions as a Key to Solve the Inconsistency of Results Concerning Performance of Subjects in Remission From Depression

The results obtained in the studies involving the QMCN strongly suggested that we should reject the hypothesis that people in remission from depression perform similarly in linguistic tasks as depressed patients. However, results of our other studies (see e.g. Bartczak and Bokus 2013; Bartczak et al. 2010) revealed a puzzling effect and inclined us not to reject the hypothesis. The same three groups of subjects (depressed patients, those in remission from depression, and non-depressed individuals) were asked to produce seven free associations for each of the five notions in the study, and for 10 randomly chosen words from the Kent and Rosanoff (1910) list. The associations provided by the subjects were then analyzed in terms of valence. Words with unequivocally positive evaluative connotations (e.g. *past*–*wonderful*, *sadness*–*friend*) were considered to have positive valence. Negative valence was assigned to associations with unquestionably negative evaluative connotations (e.g. *sadness*–*tears*, *joy*–*pointless*). Lack of evaluation (neutral valence) was assigned to items whose main function was designative (e.g., *past*–*calendar*) and to words whose axiological charge can be different for different users of language and dependent on their individual experience (cf. *future*–*job*, *past*–*pregnancy*).

The results in this case—similarly to the QMCN results—confirmed the hypothesis on the more intensive negative processing of neutral and positive notions (PAST, FUTURE, JOY, HAPPINESS) in the depressive group relative to healthy subjects. Analysis of the replies in the association task furnished one more interesting result not found in studies using other tools: a significant difference in the replies of the two non-depressive groups, that is, non-depressed individuals and those in remission from depression. These two groups differed significantly in terms of the valence of associations for PAST, and in terms of both the number of negative associations and the number of neutral and positive ones. Compared to the control group, subjects who had suffered a depressive episode in the past produced significantly more positive associations for PAST, and fewer neutral and negative ones. The high statistical significance of these differences (see Bartczak and Bokus 2013; Bartczak et al. 2010) makes them an extremely interesting problem for interpretation, especially since the result runs contrary to expectations.

The result obtained in the association task, especially after juxtaposition with the QMCN results, that did not reveal any significant differences in the two non-depressive groups’ replies, prompted us to conduct a further search. We believe that one of the possible explanations for the aforementioned discrepancy is that differences in conceptual processing between the group in remission and the control group occur especially at the first, earliest stage of conceptual processing (as understood in the LASS theory presented by Simmons et al. 2008). The LASS (*Language and Situated Simulation*) theory treats the formation of verbal associations as an important stage in the conceptual process, and associations themselves as a measure of the meaning of a given lexical unit or an indicator of the semantic relations in a given semantic field. According to this theory, the creation of associations is an essential condition for understanding notions. The LASS theory assumes that the process of understanding notions involves two intrinsic mechanisms: (a) activation in the language system and (b) situated simulation. Activation in the language system is faster: In response to a verbal stimulus, and the activation of other linguistic forms, mainly verbal associations, occur first. It is only after a given word (e.g., the word *dog*) is identified via the language system and associations are activated (e.g., *cat*, *barking*, *bone*, *meat*) that the brain starts to reproduce perceptual, motor, and mental states that are activated during interaction with the word’s referents (e.g., activation of the neuronal connections representing the look of a specific dog, contact with it, emotions it triggers). The LASS theory has been confirmed by fMRI results showing that the first stage of conceptual processing involves the activation of similar areas of the brain that are responsible for performance in the verbal association test (especially the Broca area; cf. Simmons et al. 2008).

## Problem: Research Question and Hypotheses

Since, according to the LASS theory, the first stage in understanding notions involves creating associative semantic networks, and since in our earlier studies differences appeared between the group in remission from depression and non-depressed individuals at this stage of processing of notions, we decided to investigate what semantic relations the subjects would create between the analyzed notions. Taking into account the character of depressive cognitive distortions posited by Beck’s cognitive theory of depression (1963, 1967), our focus of interest was the relation between the notions in the study and the notion of ME. Our research question was:

Do healthy subjects, depressive patients, and those in remission from depression differ in how they build semantic distances between the notions of ME and FUTURE, PAST, JOY, HAPPINESS, and SADNESS?

To keep consistency with the first part of our project (Bartczak and Bokus 2015), we wanted the notions under investigation to be conceptualized metaphorically: The participants were asked to treat them as guests coming to a party and to seat them at a round table (for details, see “Materials: The Semantic Distance Task” subsection). Our tool, the Semantic Distance Task (SDT, see below), enabled us to describe the relations between the notions as semantic distances and to express them numerically. Based on results of earlier studies (e.g., Bartczak and Bokus 2015), and on the claims of Beck’s (1963, 1967) cognitive theory of depression, we assumed that (a) compared to healthy people, depressive subjects would build greater semantic distances between ME and the notions of FUTURE, JOY, HAPPINESS, and smaller ones between ME and SADNESS, and (b) that the replies of people in remission from depression would be significantly different from those of depressive and healthy subjects in terms of the semantic distances built between the notions of ME and PAST. Considering the surprising result of the association task regarding the positive valence of associations for PAST produced by people in remission from depression, which we interpreted as an effect of defense mechanisms, we assumed PAST could be moved away from ME in the replies of those who had suffered a depressive episode in the past.

## Methods

### Participants

Three adult Polish-speaking groups participated in the study, the same subjects who had completed the QMCN and the association task. The first group (experimental group, E) comprised 30 subjects suffering from depression: patients of psychiatric wards and outpatient departments at Warsaw hospitals who were diagnosed with F32.1 and F33.1 according to the ICD-10 classification (Pużyński and Wciórka 1997; F32.1—moderate depressive episode, F33.1—recurring depression disorder, currently moderate depressive episode; cf. major depression disorder in DSM-IV; American 1994). The second group (control group, C) comprised 30 subjects who had never had depression: medical and non-medical staff from the hospitals where the study was carried out. The third group (remission group, R) comprised patients of hospital outpatient departments with a depressive episode behind them, currently in remission (with a diagnosis of F33.4 or F32 in the examination according to ICD-10; F32.1—moderate depressive episode, F33.1—recurring depression disorder, currently moderate depressive episode). The third group was the smallest one, with only 12 subjects. In the 6 months of the study it proved impossible to reach a larger number fulfilling the assumed criteria for being placed in group R.

The participants for groups E and R were chosen in cooperation with four psychiatrists. The doctors selected patients with the relevant diagnoses and then asked for their consent to take part in a study on notions. All participants gave their informed verbal consent prior to participation. Subjects were assigned to a given group based on their medical diagnosis following an in-depth interview, taking into account their result in the Beck Depression Inventory$^{2}$ (BDI; Beck 1973; Beck and Beamesderfer 1974; Beck et al. 1987; cf. also Parnowski and Jenajczyk 1977). As in other studies on depressive subjects, an individual was considered to suffer from depression if his or her result was equal to or higher than 10 (Beck et al. 1987; Ruscio and Ruscio 2002; see also Fajkowska and Marszał-Wiśniewska 2006). Group E had an average BDI value of 25.95 (range 18–48), strongly distinguishing it from the other two groups. The average BDI values in groups C and R were similar: BDI = 5.0 (range 0–9) and BDI = 5.25 (range 1–9), respectively. From among the patients with depression, seven had not suffered from depression before and 23 declared that this was their second episode.^2^

The groups were balanced for age ($\hbox {E}:M_\mathrm{age} =44.3$, range 21–77, $SD = 14.22$; $\hbox {C}:M_\mathrm{age} =44.6$, range 23–83, $SD = 17.88$; $\hbox {R}:M_\mathrm{age} =49.1$, range 23–78, $SD = 17.82$) and sex (24 females and six males in Groups E and C, and nine females and three males in group R); education, place of residence, and income per capita in the subjects’ households was also controlled.

### Materials: The Semantic Distance Task (SDT)

The SDT has not been used in earlier research projects. Its structure, based on assigning places to notions at a round table, enables the SDT to be considered a special version of the semantic distance latency test used in research on cognitive representations of different categories and notions. For example, Chiao et al. (2004) used the semantic distance technique to study representations of social status. In the “status condition”, the subjects compared the names of university occupations and ranks in the Navy with a selected anchor noun (*assistant professor *and *captain*, respectively), deciding whether a given word was of higher, lower, or of the same status compared to the anchor. In the “number condition”, there were numbers—from 33 to 99—instead of position names (the anchor was the number 65). Based on analyses of reaction times, the authors concluded that the subjects represented the names of professional ranks in the same way as distances between different numbers (comparing two positions/numbers that were far apart was faster than comparing stimuli similar to each other). The results of this study on understanding social status suggest that, in some respects, representations of semantic distance are comparable to representations of distances between numbers.

We modified the version of the task used by Chiao et al. (2004) because we wanted the notions to be understood metaphorically. Similarly to the task used by Chiao et al., the SDT was conceived in such a way that the distances between the different notions could be coded as numerical values. Our modification was that the subjects were asked to imagine that they were invited to a party attended by guests with the following nicknames: Past, Future, Joy, Sadness, and Happiness. The task of the subjects was (a) to briefly characterize each guest by listing their relevant qualities, (b) to propose two extra guests of their choice who could be invited to the party and to briefly characterize them, and (c) to seat all the guests and themselves (“me”) at a round table (the Semantic Distance Task, along with instructions for participants, can be found in “Appendix”).

### Data Analysis

**Fig. 1:**
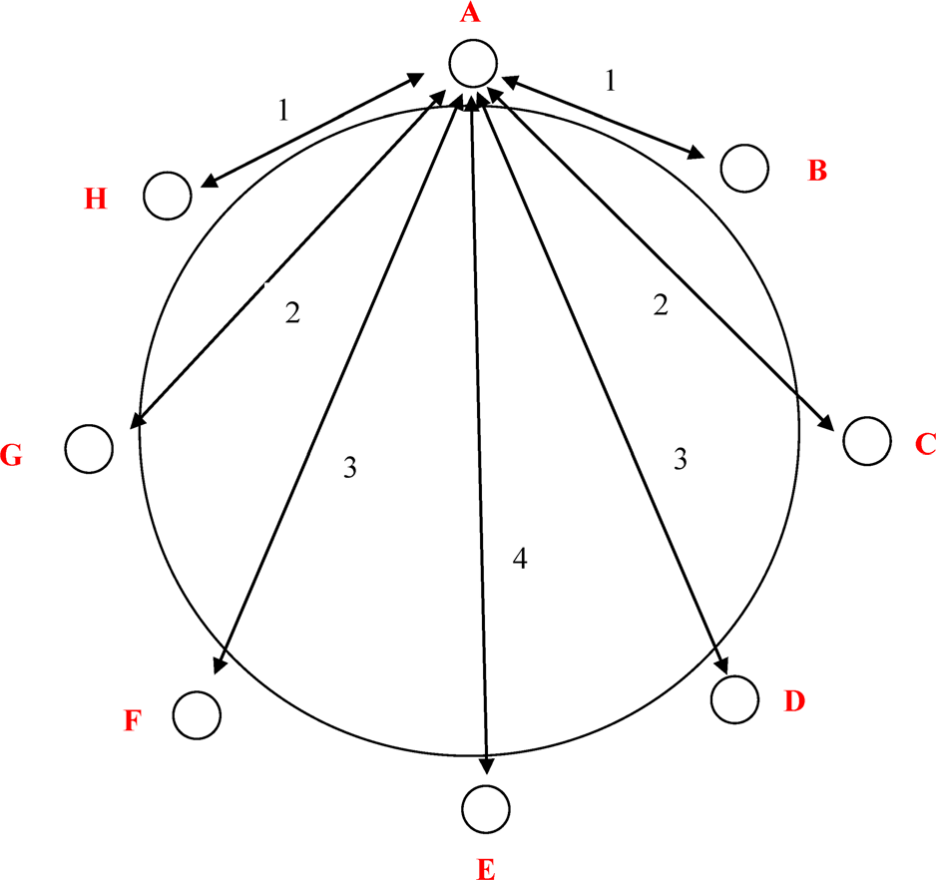
Assigning numerical values to the distances between notions, based on the results of the Semantic Distance Task

Based on the way the “guests” were seated at the table, numerical values were assigned to the distances between the different notions. This was done as follows: The distance represented by guests X and Y, seated next to each other, was given a value of 1. If guests X and Y were separated by one “person”, the value was 2; by two “people”, it was 3; and when there were three “people” in between, it equaled 4. The values assigned to the different distances are shown in Fig. 1.

Independent-measures *t* tests were used to examine the existence of significant inter-group differences in the distances between the analyzed notions and ME (for details, see the “Results” section), and an analysis using the data mining technique (with the help of the *STATISTICA Data Miner 8* software) was performed. Data mining, based on artificial intelligence elements (Nisbet et al. 2009), uses mathematical algorithms to obtain information concealed in the data and uncover its structure, and can be perceived as the method of decision-making. The program builds models illustrating the structure of the data and estimates the error rate of each model (the lower the error rate, the greater the model’s predictive power). To form a model out of a set of analyzed variables, a variable whose values are to be modeled is chosen (i.e., the target variable). This variable is explained by other categories of variables (predictors) indicated by the researcher. The aim of the analyses is to generate a mathematical function that enables the sample to be split into subgroups that are homogeneous with regard to the target variable.

There is hitherto only very limited psychological literature on data exploration using the data mining approach so far (cf. discussion in Yim et al. 2014). Papers by Chang (2007); Faulkner et al. (2010); Szymanska (2012); and Yim et al. (2014) can serve as examples.

In our research, the C&RT (Classification and Regression Tree) mathematical algorithm was used. As Szymanska (2012) describes, “the algorithm builds a decision tree graph which represents the input of the predictors in explaining the target variable.... The C&RT algorithm uses the predictors and performs the function so as to maximize the homogeneity of the groups.... The C&RT algorithm also arranges the predictors according to their importance in explaining the dependent variable (target variable) but at the same time it provides the splitting points. Such a structure of the decision tree enables the reconstruction of the differentiation, which is extremely useful when interpreting the results” (pp. 220–221).

In our model, the target variable is the nominal variable: E, C, and R, while the input variable—the distances between ME and the other notions (ME–PAST, ME–FUTURE, etc., a continuous variable).

### Procedure

All procedures were carried out according to the Declaration of Helsinki and were approved by the ethical committee of the University of Warsaw’s Faculty of Psychology. Participants were tested individually. The subjects were asked for their consent to take part in a research project on the understanding of notions. Upon consenting they were given sheets with the tools and instructions and a token of appreciation in the form of a pen with the University of Warsaw logo. Due to the increased fatigue of depressive patients, the instructions stated that the study could be interrupted if the subject felt tired, and would be continued later. The subjects filled out the questionnaire at home and brought it to their next doctor’s appointment. The directors of the units where the study was conducted agreed to patient and staff participation in the project. The selection of depressive patients for the study was made in cooperation with the psychiatrists treating them; participation in the study did not disrupt therapy in any way. We were assured by the doctors that the patients selected for the study met our selection criteria (in particular, excluding any comorbid diagnosis potentially influencing patients’ performance).

### Results

The structure of the tool enables the distances between notions to be presented in the form of numerical values. The average distances for the different notions, together with standard deviations, are shown in Table 1.

**Table 1:** Results of the Semantic Distance Task for group E (experimental), C (control), and R (remission from depression)

	PAST	FUTURE	JOY	SADNESS	HAPPINESS	PERSON 1	PERSON 2
*ME*							
E:	$$2.00\, (1.31){**}$$	2.40 (1.16)	2.07 (1.17)	2.30 (1.39)	2.17 (1.21)	1.47 (1.04)	1.53 (1.01)
C:	$2.27\, (1.11){*}$	2.73 (1.23)	2.07 (1.01)	2.80 (0.97)	1.93 (1.20)	1.50 (0.94)	1.60 (0.89)
R:	3.25 (1.21)	2.17 (1.11)	2.03 (1.16)	2.25 (1.05)	1.58 (0.79)	1.58 (1.00)	1.67 (1.07)

The data are mean distances between concepts. Standard deviations in parentheses

*C* never-depressed individuals, *E* patients suffering from depression, *R* participants currently in remission, with a depressive episode behind them

$$^{*}p < .05$$; $$^{**}p < .01$$

Looking at the results, one can see that, as expected, group E situated SADNESS a little closer to ME than group C, but in the case of both groups there were no noticeable differences of distances between ME and JOY, and between ME and HAPPINESS. However, the most interesting observations were provided by the group in remission from depression. Interestingly, in a few cases they were different from the results of both group E and C, especially as regards the temporal notions. For example, compared to the other two groups, subjects with a depressive episode behind them placed PAST farther away from ME (agreeing with our predictions), and FUTURE farther away from SADNESS, but also from HAPPINESS.

We analyzed the significance of inter-group differences in the distances between the analyzed notions and ME. The *t* Student test confirmed a significant divergence between the results of groups R, C, and E in the distance between ME and PAST. In fact, subjects in remission placed ME significantly farther away from PAST ($M = 3.25$, $SD = 1.21$) than subjects from the control group, $M = 2.27$, $SD = 1.11$, $t(40) = -2.52$, $p = .016$ (multiple comparison test LSD $p = .021$; Bonferroni adjusted $p = .06$). The effect size was large (Cohen’s $d = -0.80$). A similar difference was observed between the results of groups R and E; R: $M = 3.25$, $SD = 1.21$; E: $M = 2.00$, $SD = 1.31$, $t(40) = -2.84$, $p = .007$ (Bonferroni adjusted $p = .01$, multiple comparison test LSD $p = .004$). The effect size was large (Cohen’s $d = -0.90$). The differences between groups E and C regarding the distance to PAST, and between groups E and C, E and R, and C and R regarding the distance between ME and the other notions, were not statistically significant. The results are also confirmed by the Kruskal-Wallis nonparametric equivalent of ANOVA, $\chi ^{2}( 2 )=9.163$, $p =.010$.

In the next step, we conducted an analysis using the data mining technique, with the help of the *STATISTICA Data Miner 8* software. On a 20-% sample of results ($n = 14$) the program found the technique that was best for analyzing the given structure of the data: the Classification and Regression Trees method (C&RT; cf. Berg 2008; Nisbet et al. 2009). This technique (its structure being that of a decision tree) had the smallest error rate (14.29 %) compared to all other techniques (including Quinlan’s algorithm, Automated Neural Networks, Boosted Trees, Support Vector Machines MARSplines; see Nisbet et al. 2009), and the accuracy of the C&RT model was 85.71 %. This means that, based on the set predictors, the model could predict, with an accuracy of 85.71 %, which group (E, C, or R) a person with a given result in the SDT was from. As the three strongest predictors of group membership the program indicated the distances ME-SADNESS, ME-PAST, and ME-FUTURE. They were then used to analyze the remaining data set ($n = 58$).

**Fig. 2:**
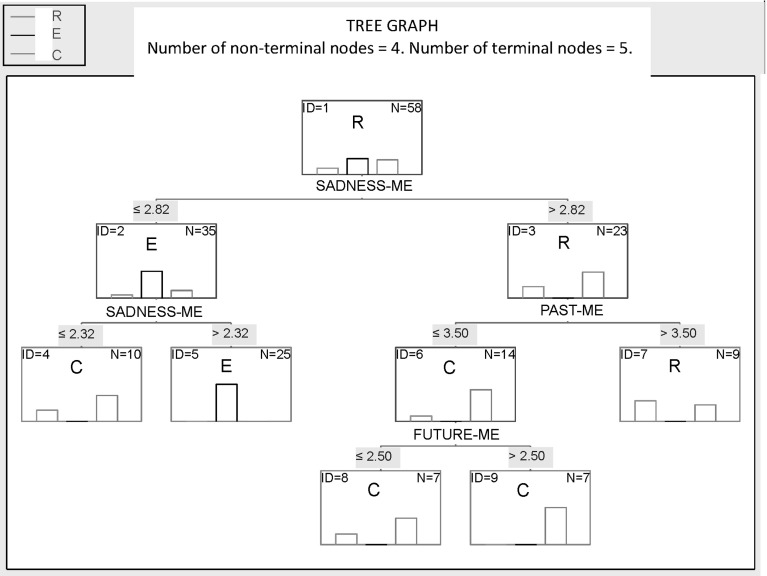
Decision tree presenting the predictors of belonging to group E (experimental), C (control), and R (remission from depression) on the basis of the ME-SADNESS, ME-PAST, and ME-FUTURE distances

The decision tree showing the predictors of the subjects’ membership in a given group is presented in Fig. 2. It turned out that the limit point dividing the sample into maximally homogeneous groups was the distance between SADNESS and ME. An average distance of over 2.82 was a predictor of a person’s belonging to the group of subjects in remission from depression, and a value lower than (or equal to) 2.82—as belonging to the depressed patient group (distance of 2.32–2.82) or to the control group (distance smaller than 2.32 or equal to 2.32). The other limit points were the distance between PAST and ME (if greater than 3.5, then group R; if smaller than or equal to 3.5, then group C) and between ME and FUTURE (if greater than 2.5, then group C or R; if smaller, then group C).

## Discussion

### Interpreting the Results

The Semantic Distance Test used in our research belongs to the group of projective techniques. It is based on a different procedure of concluding about an individual’s qualities than questionnaires, in which we ask about conscious behaviors, experiences, and beliefs (Piotrowski et al. 1993). Here, we invoke content from outside the individual’s awareness. There are no “right” or “wrong” responses. The aim is to maximize the chances of individuals revealing themselves (spontaneously, without the limitations of conscious decisions). The study involves a projection manifested in the way individuals perceive and organize the stimulatory material (a party at a round table) in terms of their own expectations, needs, goals, etc. The distance between metaphorically conceptualized notions and ME, measured by the way the guests (notions) are seated at the round table, turned out to be a device providing information on the structure of semantic networks, of which the relations between ME and PAST, FUTURE, JOY, SADNESS, and HAPPINESS are an important part.

Due to the specificity of depression symptoms, subjects without symptoms of depression were expected to place the name of a negative emotion (SADNESS) farther away from ME, and the names of positive emotions (JOY, HAPPINESS)—closer to ME than depressive subjects. The analysis results confirmed this prediction as regards SADNESS. Dividing the sample into maximally homogenous groups, the C&RT algorithms predicted that participants suffering from depression would assign a value smaller than 2.82 to the ME-SADNESS relation rather than greater than 2.82. This result is not surprising, considering that an intensified feeling of sadness is a typical symptom of depression (see e.g., Rosenhan et al. 2003, p. 272; cf. also Beck 1963, 1967, 1976; for neuroimaging evidence of a negativity bias in depression, see Fales et al. 2008).

Our second prediction regarding the SDT results was that the replies of those in remission from depression would differ significantly from the replies of depressive and healthy subjects in terms of the semantic distances built between the notions of ME and PAST. We based this supposition on the interpretation of the association task results. In the test, group R had produced more positive associations than the control group, which we interpreted as being the effect of defense mechanisms. It was interesting to observe that – true to our prediction—the greatest distances were assigned to this relation by the group of subjects in remission from depression, not by the control group. We obtained such results in both the *t* test analysis and the data mining analysis. This result, showing a significant difference between the remission group and the control group, is a unique fact for our project concerning understanding of notions in depression as a whole (Bartczak and Bokus 2013). Apart from the results of the association test for PAST, the results of the two non-depressive groups (C and R) were usually very similar and significantly different from the results of the experimental group. This makes it all the more interesting to see the significant difference in the ME-PAST distance.

One possible explanation is that the greater ME-PAST distances observed in the remitted group are the result of some kind of contrast effect or positive proactive response mechanisms. Subjects in remission, having one or more episodes of depression in the past, see the difference between their past (depressive) and present (non-depressive) functioning and highlight the dissimilarities between these two stages.

Another interpretation—as mentioned earlier—suggests that greater distances between ME and PAST built by subjects in remission are caused by defense mechanisms. *Defense mechanisms*, a notion originally referring to Freud’s (1926) theory, are unconscious psychological processes that are activated in threatening and anxiety-provoking situations (Cooper 1998; cf. Hamidi and Motlagh 2010). We speculate that a depressive episode from the past can be interpreted as a threat for the psychological and affective balance of an individual. If so, it may be that participants from the remitted group situate PAST farther away from ME, applying one of the basic psychological defense mechanisms: *denial*. A remitted individual may pretend that the threatening or anxiety-provoking situation did not happen at all. Denial is connected with overcoming depression, for example, in the well-known bioenergetic theory of depressive disorders by Lowen (1976). This theory is based on the assumption that all defense behaviors in depression are motivated by denied negative experiences from the past.

The greatest ME-PAST distance in the remitted group may also be interpreted in the light of *rationalization*, in particular, of the *sweet lemon* defense mechanism. Remitted participants may seemingly rationally explain past depressive attitudes as situationally explained and adaptive for actual good functioning. Having a depressive episode in the past may be treated as a valuable experience confronting an individual with his or her emotions, serving as a catharsis and enabling subsequent non-depressive functioning. Remitted patients may also make themselves believe that upsetting past experiences and situations were actually pleasant. The results obtained can also be explained with reference to the *reaction formation* defense mechanism that is based on creating and demonstrating attitudes and behaviors opposite to those compatible with a person’s real but consciously denied feelings.

Such interpretations are consistent with the literature on the role of defense mechanisms in the somatic and mental functioning of an individual. For example, Salimynezhad et al. (2015) provided evidence for a positive correlation between defense mechanisms and general health and emotional intelligence. Kashani et al. (2014) studied the role of defense mechanisms in predicting post-traumatic growth in cancer survivors. Hamidi and Motlagh (2010) analyzed defense mechanisms in patients with obsessive compulsive disorder and found that they use them more frequently than healthy individuals to reduce anxiety.

Importantly, there is also much evidence that defense mechanisms accompany emotional problems: depression and anxiety (Blaya et al. 2006; Perry 1988). For instance, Corruble et al. (2004) studied defense styles in depressed suicide attempters and hypothesized that defense mechanisms may discriminate between depressed patients with or without recent suicide attempts. They found that especially humor and sublimation were negatively correlated to depression intensity (cf. also Mullen et al. 1999). Stepanchuk et al. (2013), interested in coping strategies and defense mechanisms in patients with an oncological diagnosis of chronic hematological disease (often associated with depressive symptoms and anxiety), found that the interaction of coping strategies with the defense mechanisms of escape/avoidance and denial favors adaptive behavior.

Summing up, defense mechanisms may soften anxiety, frustration, and feelings of guilt evoked by past depression, enhancing non-depressive functioning of an individual. As asserted by Cramer (1998), the neurotic defense mechanisms (e.g., *rationalization* and *reaction formation*) are usually adapted to protect a subject from experiencing unacceptable thoughts or feelings. Defense mechanisms can aid individuals’ therapy of depressive disorders, helping the patients to face psychological and affective changes (Bond and Perry 2004). This explanation is cohesive with the interpretation of the other significant difference between groups R and C found in our study. In the association task (for a detailed description of the results, see Bartczak and Bokus 2013; Bartczak et al. 2010), subjects in remission from depression produced associations with PAST with a higher valence than the control group. This result was also discussed, among other considerations, in terms of defense mechanisms serving to assimilate a depressive episode from the past.

Another relation that distinguished the three groups was the ME-SADNESS relation. In accordance with our prediction, patients suffering from depression and remitted individuals assigned smaller semantic distances to this relation than never-depressed participants. The result concerning depressive people comes as no surprise and is compatible with clinical and theoretical descriptions of depression symptoms. The results of the remitted group are in agreement with our hypothesis that negative bias in understanding notions would be observable during remission of the disorder. We propose to treat this result as confirmation of the prediction that intergroup differences would be apparent, in particular, in the first, associative stage of understanding notions (cf. the LASS theory). This would also explain the unexpected results of the first part of our study (Bartczak and Bokus 2015) where the performance of the remitted group was just like that of never-depressed individuals. The interpretation of this fact might be that in the first part of the study, the problem under investigation was processing of metaphorical sentences, while in the SDT, we concentrated on an earlier, more basic stage of conceptual processing: creating associations and networks between metaphorically conceptualized notions.

The Semantic Distance Test can also be considered (Ważyńska et al. 2015) as a way of measuring the distance between different I-positions (different points of view available to a person) according to the concept of the dialogical self of Hermans (1999). Each potential I-position constitutes a separate perspective for the perception and interpretation of experience (Hermans 2002). The agentive self can move between the different I-positions, here: positions occupied by personified metaphorical notions—guests at a round table. These positions, having not only a certain vision of the world but also a voice (as they do in Bakhtin 1984), are potential participants in internal dialogues. The agentive self can choose specific interlocutors more often or less so, it can conduct a cooperative dialogue with guests sitting nearer or a confrontational one with guests sitting farther away at the round table. The level of tension within the dialogical self can be of varying degree. Sometimes the affective state, the cognitive perspective of a given I-position creates tension and is pushed away by the agentive self which even resorts to defense mechanisms. Our research seems to suggest differences within the dialogical self in patients with depression and those in remission from depression.

## Conclusions

In summary, SDT has emerged as a tool that differentiates the replies of all three groups in the study. Based on the results, we can offer the following conclusions:

1. A negativity bias in depression also occurs at the level of understanding notions. Our results show that depression-related cognitive changes described by cognitive theories of depression, including Beck’s (1963, 1967) theory of depression, occur not only at the level of thinking, and are not related exclusively to the creation of dysfunctional thinking patterns, but are also observed at the notion comprehension level, especially for notions that are key from the point of view of depression symptoms (PAST, FUTURE, and SADNESS).
2. The first stage of understanding notions, connected with the formation of semantic associative networks (cf. the LASS theory; Simmons et al. 2008), seems especially interesting as a research problem. It is only in tasks requiring the creation of semantic associative relations that statistically significant differences were observed between the replies of all three groups in the study: not only the group of depressed subjects and the group of non-depressed individuals, but also the group in remission from depression.
3. PAST (and perhaps also SADNESS) seems to be a particularly important notion from the point of view of studying conceptual processing in depressive subjects and those in remission from depression. The results obtained in tasks requiring processing of this notion significantly differentiated the performance of these groups. At the same time, they could suggest that the depressive cognitive triad (ME-FUTURE-WORLD) should perhaps be supplemented with this notion as another area in which patients currently suffering from depression, and those who suffered from it in the past, concentrate their thoughts.

### Limitations of Results and Goals for Further Research

The main limitations of the present results are the medications taken by members of the experimental group, the lack of any assessment of the participants’ verbal intelligence, and the specificity of the selection of subjects for the depression remission group.

The members of the experimental group were chosen from among patients of hospital psychiatric wards and outpatient departments. For obvious reasons, it would have been unethical to have them stop using their medication during the study. One needs to remember that the people suffering from depression were taking antidepressants at the time. These drugs relieve the symptoms of depression, and there is also evidence of their positive impact on cognitive processes (for a review, see Talarowska et al. 2009). Therefore, one can accept with a high level of probability that the medication could have affected the depressive patients’ performance of the tasks in the study.

The lack of control of verbal intelligence in all three groups of participants is another important limitation of the study. Because of the increased fatigue of patients suffering from depression, we decided not to include another tool, e.g. the WAIS-R(PL) vocabulary subscale (Polish adaptation of the Wechsler scale, Brzeziński et al. 1996), to measure verbal intelligence. Instead, all three groups were balanced for education. We assumed that education is highly correlated with vocabulary and, generally, greater cognitive reserve, due to usually better synaptic connections and more effective use of alternative cognitive strategies in educated individuals (cf. remarks in Kahlaoui et al. 2012, a study on verbal fluency). However, in doing so, we cannot clearly determine if any obtained differences are due to verbal intelligence or not. A similar problem arises with the lack of normalized indicators of general cognitive functioning (e.g., performance in the Mini-Mental State Examination, MMSE or Montreal Cognitive Assessment, MoCA). Instead, in our project on understanding notions in depression, the number of replies not given by a subject in the association task was taken as a simple measure of cognitive function disorder. This was also caused by the specificity of the depressive group under study. Our goal was to make the methods maximally easy and quick to use, but the consequences include limitations in the generalization of the study results.

Another major limitation in generalizing the results is the specificity of the group of patients in remission from depression (R). During study participant selection, the criteria for a subject to belong to this group included (a) having had a depressive episode in the past and (b) current remission of depression (i.e., having no symptoms of depression in the BDI) during the study. This condition made it rather difficult to find participants for group R. Most of the patients seeing doctors for checkups, diagnosed with “recurring depressive disorders, currently in remission” achieved results in the Beck Depression Inventory that indicated a depressive state. Over several months of the study, working with four psychiatrists, we managed to find 12 subjects meeting the above criteria. This still leaves unanswered the question of how representative the sample was, and whether the members of group R were typical representatives of the group in remission from depression. The criteria of participants’ selection were stringent and this may have significantly influenced the results.

Testing partially-remitted individuals is an important goal for future research. On the other hand, there is clearly a need to find a more precise definition of the *remission of depression* in diagnostic standards. Do we define *remission* as the complete disappearance of symptoms, or a significant subsiding of their intensity that is perceptible to the patient? Although there are proposals in the literature of what *remitted* means (e.g., Frank et al. 1991, suggest diagnosing remission if a patient scores less than 7 in the Hamilton Rating Scale for Depression (Hamilton 1980) and manifests no depressive symptom severe enough to impair function within 3 months), many authors stress that *remission* is still variously defined (e.g., Manber et al. 2008). Resolving these factors may prove to be the key to interpreting the contradictory results of research on the lasting nature of cognitive changes during remission from depression.

The results discussed here could provide inspiration for expanding the cognitive theories of depression originating from the ideas of Beck (1963, (1967). First of all, they prove there is a need for cognitive models of depression to include a conceptual level of disorders (i.e., one associated with understanding notions). To our knowledge, the problem of conceptual process disorders in depression is seldom included in contemporary research projects (for more details, see Bartczak 2009; Bartczak and Bokus 2013, 2015). The issue is also ignored by theoretical concepts explaining the etiology of depression, e.g., the best known cognitive theory of depression—Beck’s theory of negative cognitive patterns—focuses mainly on disturbances at the mental process level, not at the conceptual level. Listed examples of cognitive distortions include excessive generalization and dichotomous thinking, and not disturbed cognitive representations of PAST or HAPPINESS.

Researchers seem to have shown the greatest interest in the perception of temporal notions and the way depressed subjects sense time. However, in contemporary research it is hard to find projects dealing strictly with the way depressed people understand different concepts. There have been some studies comparing perception of the passage of time in patients with depression and healthy subjects (cf. the time reproduction task in Mahlberg et al. 2008; see also studies by Gil and Droit-Volet 2009, and by Sévigny et al. 2003). However, these studies do not touch upon depressive subjects’ comprehension of notions related to time, but only the perception of time itself.

Our studies are a modest contribution on how patients with depression, and those in remission from depression, understand notions. This research problem has yet to be fully explained and should be addressed by future interdisciplinary studies. Considering the results we have obtained, tools requiring the creation of semantic associative relations seem the most promising, because it was only at this stage of conceptual processing that we managed to identify differences between the performance of those in remission from depression and non-depressed individuals. The question remains as to why processing of only some of the notions brought significant inter-group differences. Perhaps this is related to the semantics of the stimuli as a factor with a significant impact on directing the attention and on the course of cognitive processes in depressed patients (cf. the content-specificity hypothesis; Beck 1976). The problem of how depressed subjects, and those in remission from depression, process notions still requires much research before it becomes possible to formulate any firm conclusions regarding the distortion of conceptual processing caused by depression.

## Appendix: The Semantic Distance Task

Imagine that you have been invited to a party. When you arrive, you discover that the guests include five people with the following nicknames: Past, Future, Joy, Sadness, Happiness.

1. What do you imagine these guests are like? Please characterize these people briefly by writing their relevant qualities:
2. What other two people would you like to take to the party with you?
3. Please characterize these people briefly:
4. Where at the table would you sit at the party? Where would you like the other five people to sit, and the two people that you think are missing from the guest list? Please label the seats around the table accordingly.

## Footnotes

1 In many sources studying depression symptoms, these particular terms are used for describing cognitive distortions and affective disorders in depressed patients. Among other things, it is believed that depressive subjects perceive their past chiefly as a source of failure and paint their future in dark colors, that the dominating mental state of depressed patients is sadness, and that patients are unable to feel joy or to be happy (e.g., Rosenhan et al. 2003, p. 272; cf. also descriptions of depressive symptoms in ICD-10, Pużyński and Wciórka 1997; and in DSM-5). The same notions are also used to characterize symptoms of depression in theoretical models of depressive disorders, e.g. Beck’s (1963, 1967) cognitive theory of depression, or Clark and Watson’s (1991) tripartite model of anxiety and depression.

2 The Polish translation of the BDI was used; when we were conducting our research, research on the Polish adaptation of the BDI-II was being conducted elsewhere (see e.g., Zawadzki et al. 2009).
